# A Hybrid Model for Driver Emotion Detection Using Feature Fusion Approach

**DOI:** 10.3390/ijerph19053085

**Published:** 2022-03-06

**Authors:** Suparshya Babu Sukhavasi, Susrutha Babu Sukhavasi, Khaled Elleithy, Ahmed El-Sayed, Abdelrahman Elleithy

**Affiliations:** 1 Department of Computer Science and Engineering, University of Bridgeport, Bridgeport, CT 06604, USA; susukhav@my.bridgeport.edu (S.B.S.); ssukhava@my.bridgeport.edu (S.B.S.); aelsayed@bridgeport.edu (A.E.-S.); 2 Department of Computer Science, William Paterson University, Wayne, NJ 07470, USA; elleithya@wpunj.edu

**Keywords:** convolutional neural network, hybrid model, driver emotion detection, ADAS (advanced driver assistance systems), facial expression recognition, machine learning, support vector machine

## Abstract

Machine and deep learning techniques are two branches of artificial intelligence that have proven very efficient in solving advanced human problems. The automotive industry is currently using this technology to support drivers with advanced driver assistance systems. These systems can assist various functions for proper driving and estimate drivers’ capability of stable driving behavior and road safety. Many studies have proved that the driver’s emotions are the significant factors that manage the driver’s behavior, leading to severe vehicle collisions. Therefore, continuous monitoring of drivers’ emotions can help predict their behavior to avoid accidents. A novel hybrid network architecture using a deep neural network and support vector machine has been developed to predict between six and seven driver’s emotions in different poses, occlusions, and illumination conditions to achieve this goal. To determine the emotions, a fusion of Gabor and LBP features has been utilized to find the features and been classified using a support vector machine classifier combined with a convolutional neural network. Our proposed model achieved better performance accuracy of 84.41%, 95.05%, 98.57%, and 98.64% for FER 2013, CK+, KDEF, and KMU-FED datasets, respectively.

## 1. Introduction

Basic transportation all over the world still relies on automobiles. To support this, the American Automobile Association (AAA) foundation for traffic safety [1] provided a report on the USA in 2019, stating that the average time spent driving per driver is about an hour every day with a road coverage of 31.5 miles, which is a 5% increase when compared with 2014 statistics. In 2019, Americans spent 70 billion hours driving, which is 8% more than in 2014. This increase in time spent driving shows that the car could be seen as an ambient living space for the people driving on roads. Driver-related risk factors [2] such as cell phone distraction, alcohol consumption and aggressive driving influence the driver’s behavior. The American Automobile Association (AAA) foundation for traffic safety on driving behavior [3] in 2020 reported that 37% of the people are distracted when driving due to texting or reading on a cell phone, 7% are consuming alcohol, and 45% of people are driving over the speed limit on freeways, affecting the safety of pedestrians. Pedestrian safety [4] is a global major concern, and to lower mortalities, many improvements are being made to build a safe walking environment for vulnerable road users, especially older people, to keep them away from exposure to aggressive people driving automotive vehicles on roads. Along with major road accidents caused by aggressive driving behavior, around half of the injured drivers in minor road accidents were also not fully recovered and prone to slow recovery, which takes them away from work and regular human activities.

Emotions are the factors that influence a driver’s capabilities towards either the positive side or negative side of driving. To monitor the driver’s emotions, face expression recognition technology is used to detect the face and expressions of the person. Facial expression analysis is creating a positive impact on the development of human machine interactions for safe driving behavior and road safety. Expressing emotions [5] usually happens in two methods of communication, namely, verbally and non-verbally. Verbal communication is easy to communicate and understand between the people in most situations, whereas nonverbal communication, such as showing emotions, is difficult to understand in some situations.

A person’s mental state can control a person when going through either a good situation or a bad situation, especially if the driver’s emotions are considered. Emotions such as happiness and neutrality can put the driver in a good mental state and able to drive the vehicle safely. However, emotions such as sadness, anger, disgust, and fear influence the driver’s capabilities to cause road accidents. To avoid this, driver emotion monitoring [6,7] became a crucial and necessary module in the advanced driver assistance systems [8] in most automotive vehicles. These systems will control the vehicle functions according to the driver’s emotions and help in avoiding road accidents.

Driver emotion detection in advanced driver assistance systems (ADAS) is accomplished by using facial expression recognition (FER) technology. FER is achieved by deep neural networks [9] alone or using a hybrid model involving SVM as a classifier to convolutional neural network (CNN) or using manual feature extraction methods with machine-learning-based classifier implementations. Most of the developed works so far have chosen one of these directions to perform face expression recognition. However, several factors affect these methods, from predicting the proper class of expressions and attaining high accuracy. Our proposed approach is novel in creating a hybrid model with a dedicated pre-processing stage to handle illumination conditions and noise removal. Features are extracted manually using Gabor and LBP methods. These extracted features are fused and fed to CNN, providing more robust features given to SVM for classification. Our objective is to handle the major challenges such as pose variations and occlusions in face expression recognition. We successfully achieved our objective by achieving better accuracy in predicting emotions from the faces involving these challenges, compared with state-of-the-art methods. The remainder of the paper is organized as follows: Section 2 reviews the related works. Section 3 presents the proposed hybrid model architecture. Section 4 presents experimental results. In Section 5, the conclusions are offered.

## 2. Related Works

Many developments were made to design an efficient advanced driver assistance system, which is the main domain in autonomous vehicles. One of the main functions of ADAS is to monitor and detect the driver’s emotions, which helps track the driver’s health conditions and emotions and thereby avoid auto road accidents. Driver emotion monitoring and detection were accomplished in three directions: creating driving patterns from travel and driving history, collecting the physiological signal data, and inside inbuilt vehicle-camera-captured image analysis. The first direction predicts the driver’s methodology from his or her movements of steer wheel usage, lane change driving behavior, and break time usage while driving in real-time. It is not an accurate methodology due to its inadequate road route data that vary differently from place to place.

The second direction utilizes the information from physiological signals such as electrocardiogram (E.C.G) [10], electroencephalogram (E.E.G) [11], photo plethysmography (P.P.G) [12], electrical dermal activity (E.D.A) [13] by connecting physical sensors to the human body [14] that is difficult to focus on driving due to these body-worn sensors. To overcome this problem, the third direction is evolved by analyzing the image captured by the camera sensor inside the vehicle to track the driver’s emotions without any physical contact. Usually, facial expression recognition is the most crucial in detecting driving anomalies and avoiding the passengers having car sickness from their facial expressions. Hence, we have focused on designing an efficient driver facial expression monitoring system that can track the variations in driver emotions such as happiness, fear, surprise, sadness, disgust, neutrality, and anger. These are the seven standard expressions usually chosen to track inside the vehicle during driving. Generally, the conventional facial expression recognition method is made either by geometrical features evolved from the relation between the facial components [15] or by appearance features from global faces. Geometric features explain the location of the facial components, and the features involve the geometrical relationships among the feature points, which can help generate a feature vector.

Appearance features explain the face pattern with the help of different feature descriptors such as local binary patterns [16], Gabor filters [17], etc., to extract the features from an image. Pre-trained classifiers such as support vector machines (SVM) [18], RF’s [19], and KNN [20] are used to recognize the expression from the extracted features. SVM is one of the strongest classification methods and supervised machine learning techniques with four kernel types to yield better performance. Recently, deep learning (DL) approaches explicitly convolutional neural networks (CNN) [21] became more prevalent in feature extraction and classification tasks. From the collected literature, it is observed that FER models are implemented in three categories, namely, conventional machine-learning (ML)-based methods, deep-learning (DL)-based methods and the hybrid models that combine both ML and DL methods.

Firstly, the machine-learning (ML)-based methods, including those detailed by M. Jeong et al., 2018, [22] proposed driver facial expression recognition in real-time for safe driving using weighted random forests with and without hierarchy, and achieved an accuracy of 92.6% on CK+ dataset. S. Yasmin et al., 2020 [23] proposed an improved facial expression recognition using robust multiscale featured local-binary-pattern-based feature extraction combined with SVM classification, obtained 89.0% accuracy on the KDEF dataset. J. Nam et al., 2020 [24] proposed a light-weight multi-layered random forest classification model involving the combination of angel and distance ratio feature vectors, which achieved an accuracy of 93.4% on CK+ dataset. Ben. Niu et al., 2021, [25] proposed an algorithm with the combination of oriented fast and rotated BRIEF (ORB) features and local binary patterns (LBP) with SVM as a classifier, achieved an accuracy of 93.2% on the CK+ dataset. G.V.M. Vijaya Lakshmi et al., 2021, [26] proposed a method that merged spatial pyramid Zernike-moment-based shape features and law’s texture features to predict the micro and macro details of each expression, and achieved an accuracy of 88.8% with a radial basis function feed forward artificial neural network on the KDEF dataset.

Secondly, with the rapid developments in deep-learning (DL)-based methods including S. Xie et al., 2018, [27] proposed deep comprehensive multi-patch aggregation convolutional neural networks using hierarchical features which include both local and holistic features for FER. This achieved an accuracy of 93.46% on the CK+ dataset. P V Roshini et al., 2018, [28] proposed a multi-convolutional neural network approach for face expression recognition with four pre-trained CNNs and achieved an accuracy of 89.58% on the KDEF dataset. R.P. Krishna et al., 2019, [29] proposed the usage of the gradient and laplacian outputs of input images together with the actual input image given to CNN, which helped in recognizing the facial expression and achieved an accuracy of 88.16% on the KDEF dataset. A. Agarwal et al., 2020, [30] proposed CNN models with hyper-parameter selectivity and achieved an accuracy of 65% on the FER 2013 dataset. M.N. Riaz et al., 2020, [31] proposed a new CNN called “expression net” (eXnet) which has a parallel feature extraction mechanism, achieving an accuracy of 74% on FER 2013 dataset. S. Minaee et al., 2021 [32] proposed a DL framework for detecting the salient regions of interest where most of the features will exist that help in classifying expressions with an accuracy of 70% on the FER 2013 dataset. S.J. Park et al., 2021, [33] proposed a robust facial expression recognition algorithm based on a multi-rate feature fusion scheme on multi-depth CNN, achieving an accuracy of 96.23% on the CK+ dataset. S.H. Abdullahi et al., 2021 [34] proposed a deep-learning-based FER system with a label-smoothening mechanism, achieving an accuracy of 96.6% on the KDEF dataset.

Lastly, the approaches that combine deep neural networks and machine learning algorithms form hybrid models to yield better performance. B. Hasani et al., 2017, [35] proposed a 3D CNN architecture with 3D inception residual network and LSTM (long short-term memory) to extract spatial and temporal relations within a facial image, achieving an accuracy of 93.2% on the CK+ dataset. G.I. Mariana et al., 2018, [36] proposed an automatic feature learned approach using CNN and a bag of visual words model computed with handcrafted features with an SVM classifier for facial expression recognition, obtaining an accuracy of 75.4% on the FER 2013 dataset. Li. Ch et al., 2018, [37] proposed a multi-network fusion-based CNN with SVM as a classifier, achieving 70.3% accuracy on the FER 2013 dataset. A.R. Garcia et al., 2018, [38] proposed a new hybrid deep learning emotion recognition model with a DL network for self-learnt feature extraction; SVM is used for emotion classification, achieving 96.26% accuracy on the KDEF dataset. T. Cao et al., 2019, [39] proposed a FER model that introduced the K-means clustering idea and SVM classifier in the CNN framework, achieving an accuracy of 80.3% on the FER 2013 dataset. S. Liu et al., 2020, [40] proposed an improved CNN combined with SVM to perform facial expression recognition in which a pre-trained network called “VGG11” was considered to extract features from an input image and fed them to SVM for classification, obtained an accuracy of 92.2% on CK+ dataset. Z. Fei et al., 2020, [41] proposed a deep convolutional neural-network-based emotion analysis framework that is able to process facial images and interpret the temporal evolution of emotions among them with LDA classifier, obtaining 88.4% accuracy on the KDEF dataset. Lie. Yang et al., 2021, [42] proposed a transfer learning method formed by merging the inception and residual neural network “IRCNN” with SVM as classifier for facial expression recognition, achieving an accuracy of 68.1% on the FER 2013 dataset. Y.K. Bhatti et al., 2021, [43] proposed a FER approach using feed-forward learning model, in which deep features are extracted using multiple CNNs; the regularized extreme learning machine was used for classification, achieving an accuracy of 86.5% on the CK+ dataset.

Using efficient face expression recognition systems, many works developed driver emotion monitoring, including the study conducted by M. Jeong et al., 2018, [22] who proposed driver facial expression recognition in real-time for safe driving using weighted random forests with and without hierarchy, achieving an accuracy of 94.0% on the KMU FED dataset. J. Zhang et al., 2019 [44] proposed a connected CNN that combines low-level and high-level features to train deep networks for FER, achieving an accuracy of 97.3% on the KMU FED dataset. Jaeyeal Nam et al., 2020 [24], proposed a driver emotional status monitoring system using lightweight multilayer random forests and achieved an accuracy of 95.1% on the KMU FED dataset; they also conducted comparative experiments on deep-random-forest-based methods such as FTDRF (forward thinking deep random forest), which consists of a cascade forest model and deep random ferns [24] based on multiple random fern-layered structures without back propagation, achieving an accuracy of 93.6% and 91.2% on the KMU-FED dataset. They also conducted experiments on state-of-the-art deep neural networks such as SqueezeNet [24], MobileNetV3 [24] which are pre-trained and achieved an accuracy of 89.7% and 94.9%, respectively, on the KMU-FED dataset. A. Leone et al., 2021, [45] proposed a driver’s road rage detection frame with facial expression recognition using “VGG16”, which achieved 94.27% accuracy on the KMU-FED dataset.

From all the works, it has been observed that the existing face expression recognition systems efficiently detect face expressions in normal living conditions. Real-time driving conditions, including illumination conditions, occlusions, and angular poses, will affect the system to attain the highest accuracy. We proposed a novel hybrid model architecture for facial expression recognition to solve this problem. Its efficiency is improved by introducing a framework with three parts: pre-processing, segmentation, and feature extraction before the hybrid classification model. Firstly, noise removal, image enhancement, and restoration processes will be performed pre-processing. AdaBoost learning algorithm [46] is used for face location detection, and Haar features [47] are used to further identify and segment the frontal facial features, including the left eye, right eye, nose, and mouth. Secondly, Gabor [17] and LBP feature extraction methods [16] are used to extract the necessary features for facial expressions, and feature fusion of these methods is fed to the hybrid classification network where the SVM [18] is added as the classifier to the convolutional neural network [21].

We have selected the benchmark datasets FER 2013 [48], CK+ [49] for multiclass face expression recognition of different ages, KDEF [50] for five different angular subjects with multiclass facial expressions, and the KMU FED [51] dataset for the real-time driving environment with challenges such as illumination conditions and occlusions. Training our hybrid network on these datasets helped to overcome the current challenges in detecting the driver’s behavioral emotions.

## 3. Proposed Hybrid Model Architecture

**Figure 1:**
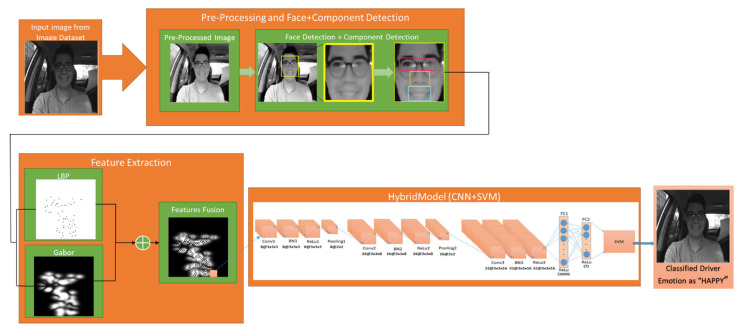
Hybrid Network.

A novel hybrid approach is proposed to detect the driver’s emotions, as shown in Figure 1. The first block in our hybrid model is pre-processing. The methods explained below are used to perform image resizing, noise removal, and image enhancement on a given input image to be fed to face detection with a minimum error rate.

### 3.1. Image Pre-processing

#### 3.1.1. Image Resizing

Image resizing can be implemented using the nearest-neighbor interpolation method [52]. It is the fastest and simplest implementation technique to scale an image. It replaces every pixel with the next nearest pixel and will provide a smoother image at the output. We have resized the input images to 256 × 256 pixels.

#### 3.1.2. Noise Removal

In this stage, we have used a 2D Gaussian filter [53] to generate blur in a scaled input image. The Gaussian filter works on a standard deviation of 0.5 and can be represented with an equation

(1) $$g\left(i,j\right)=e^{\frac{-\left(i^{2}+j^{2}\right)}{2\sigma^{2}}}$$

where (*i*, *j*) are the coordinates from origin to the horizontal and vertical axis. The concentric circles’ surface contours are generated with a Gaussian distribution from the center point. When the Gaussian filter size of 3 × 3 is applied to a noisy image, it will smoothen the image and reduce noise. It has many advantages of being a small filter. It suppresses the noise to a maximum extent. As the size of the filter increases, the Gaussian filter starts suppressing intensities occupying many pixels until the surrounding edges become smoothened.

#### 3.1.3. Median Filter

This filter adopts a nonlinear method [53] for noise removal from scaled input images. It works by sliding pixel by pixel, replacing each pixel value with the median value of the neighboring pixels. The window pattern with a size of 3 × 3 is used to slide pixel by pixel over the neighbors in a scaled input image. The median calculation can be carried out by sorting all the pixel values initially in numerical order in window pattern and replacing the values of the pixels with the middle pixel value.

#### 3.1.4. Histogram Equalization

This method is applied to improve the contrast of the scaled input image by using its histogram. This process [54] can be performed by distributing the pixel intensity values of an image which are frequently occurring and thereby low contrast areas of the image will gain higher contrast.

The histogram equalization h of a given image can be calculated by

(2) $$\;g_{i,j\;}=floor\left(\left(L-1\right)\sum_{n=0}^{f_{i,j}}p_{n}\right)\;$$

where *floor* () rounds the value to the nearest integer and $p_{n}$ is the normalized histogram of a given image *f* which pixel intensities range from 0 to L − 1. *L* is the number of possible intensity values.

#### 3.1.5. Wiener Filter

It performs two-dimensional adaptive noise removal filtering [55] with a window size of 3 × 3 that removes the blur generated during Gaussian smoothening. Therefore, the image will be restored without noise.

The local mean and variance of each pixel can be calculated by

(3) $$\mu =\frac{1}{NM}\sum_{n_{1},n_{2}\in \vartheta }\alpha \left(n_{1},n_{2}\right)$$

(4) $$\sigma^{2}=\frac{1}{NM}\sum_{n_{1},n_{2}\in \vartheta }\alpha^{2}\left(n_{1},n_{2}\right)-\mu^{2}\;$$

(5) $$b\left(n_{1},n_{2}\right)=\mu +\frac{\left(\sigma^{2}-\beta^{2}\right)\;}{\sigma^{2}}\left(\alpha \left(n_{1},n_{2}\right)-\mu \right)$$

where ϑ is the *N* × *M* local neighborhood of each pixel in a scaled input image and $\beta^{2}$ is the noise variance.

### 3.2. Face and Facial Components Detection

**Figure 2:**
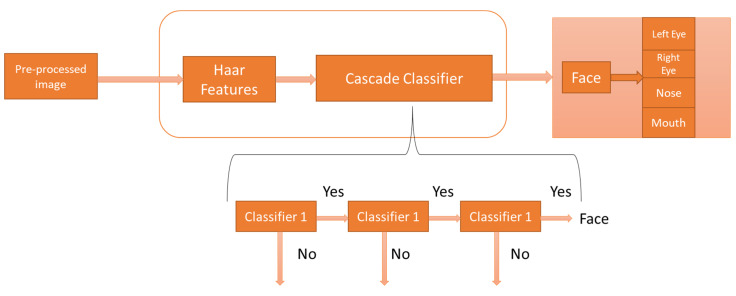
Face and face components detection using Haar features cascade classifier.

This process involves facial parts recognition from the detected face using the training of accurate classifiers with AdaBoost learning algorithm [46], followed by the cascaded classifier structure shown in Figure 2. The integral image is used for efficient computation from the sum of values of a pixel grid. The integral image at location (*a*, *b*) is represented as *I*($\hat{a},\;\hat{a}$).

(6) $$I(\hat{a},\hat{b})=\sum_{\hat{a}\leq a,\hat{b}\leq b}i\left(a,b\right)$$

where *i*(*a*,*b*) is the input image.

This algorithm is evolved from the object detection framework, and its primary purpose is to detect the face with high accuracy and less computation time. This is the best algorithm for real-time applications, but it takes more training time. This algorithm [47] operates in two steps, namely, detection and training. During the detection stage, conversion of the input image to greyscale image and face will be detected on the greyscale image using a box search on the image. To search the face in the image, Haar-like features are used. Since human faces contain similar features, Haar-like features search for similarities by creating three types of Haar features: edges, four-sided features, and line features on the face.

An integral image is made by using these Haar features, and it also makes the model run faster, as well as reducing the computation cost. A base window size of 24 × 24 is used for the Haar features. In the training stage, a boosting algorithm called the AdaBoost learning algorithm is applied to choose a few prominent features from the large set to perform efficient detection. After the learning process, a cascade classifier is allocated to reject non-face images depending upon the absence of prominent facial features selected by the AdaBoost learning algorithm. Adaboost learning is used for learning, and this algorithm builds a strong classifier with a combination of weak learners with weights, cascading these classifiers into an architecture for equal computation. Usually, cascading the more complex classifiers yields better detection. Due to this, the evaluation of strong classifiers which are developed by a learning algorithm can be processed quickly.

In the cascaded architecture, each successful classifier will be trained with the selective samples only and proceed them to the preceding classifiers. If any classifier rejects the samples at any stage, no further processing will be performed with those samples. The first classifier in this cascaded architecture is called an attentional operator, which utilizes two features. The cascade classification models are trained with image sizes of (12 18), (15 18), (15 25) for detecting the facial features such as eyes, nose, and mouth, respectively, with a scale factor of 1.1 and detection threshold value of 4 that is defined to declare a final detection in an area where multiple detections are made. A large threshold value helps in suppressing false detections, and the corresponding R.O.I. (regions of interest) are extracted and highlighted.

### 3.3. Feature Extraction

Bio-inspired convolutional kernels are extensively used in the image processing domain to solve computer-vision-related problems. Selectivity with orientation and location frequency are two remarkable characteristics of this kernel. Therefore, using these filters to analyze the images are useful, particularly in texture representation and recognition. Gabor filters [17] have different orientations and scale values to create a Gabor filter bank. Usually, the Gabor filter bank has eight orientations and five scale values. Among those, the wavelength of 4 with 90° orientation is selected for our model. These filter banks are used to perform feature extraction from grayscale images. The main advantage of the Gabor filter is that it can simulate the visual cortex receptive field compared with the remaining edge detectors. It performs the best localization in spatial and frequency domains by analyzing the signal uncertainty in spatial frequency and orientation.

We have used the fusion of Gabor filters, and linear binary patterns (LBP) features in our model. Gabor filters successfully implement texture classifications, character recognition, fingerprint recognition, and face recognition. It is the best method due to its optimal localization functionalities in the spatial and frequency domain, and it consumes very little computation time.

The spatial domain impulse response and the frequency domain response of the 2D Gabor filter are:

(7) $$h(x,y;f,\theta )=\frac{1}{\sqrt{\pi \sigma^{1}\sigma^{2}}}\exp(-\frac{1\;}{2}\left(\frac{R_{1}^{2}}{\sigma_{1}^{2}}+\frac{R_{2}^{2}}{\sigma_{2}^{2}}\right)).\exp\left(i\left(f_{x\;}x+f_{y\;}y\right)\right)$$

where *R*_1_ = *x* cos θ *+ y* sin θ, *R*_2_ = *−x* sin θ *+ y* cos θ, *σ*_1_ = *c*_1_/*f*, *σ*_2_ = *c*_2_/*f*, $f_{x\;}=f$ cos θ, $f_{y\;}=f\;\sin\;\theta ,\;$*c*_1_ and *c*_2_ are two constants.

The coefficient $\sqrt{\pi \sigma_{1}\sigma_{2}}$ represents the various Gabor filter energies which are all equal to one, that is ||h||^2^=∬ hh∗dxdy=1.

(8) $$H(u,v;f,\theta )=2\;\sqrt{\pi \sigma_{1}\sigma_{2}}\;\exp\;(-\frac{1\;}{2}(\sigma_{1}^{2}{\left(s_{1}-f\right)}^{2}+\sigma_{2}^{2}s_{2}^{2}))$$

where $s_{1}=u\cos$θ + v sinθ, $s^{2}=-u\sin\theta$ + vcosθ.

#### Linear Binary Pattern

It is an efficient texture pattern descriptor [16] to define local texture patterns, widely used in image processing applications. Its block size is 3, and the center pixel is used as the threshold pixel value for all the neighboring pixels. It compares the sample pixel of a neighboring window with the center pixel and will generate a corresponding binary code that encodes the local behavior of a grayscale image. By concatenating all these binary codes in a clockwise manner starting from the top left neighbor, the final decimal value of the binary code replaces the center pixel value at the end.

It is an efficient feature extraction method used for face detection and pattern recognition applications. The operator is a linear binary pattern that converts an input image to an array of integer labels containing small amounts of equally distributed information of an image. Different steps are involved in LBP feature extraction:

(*i*) *Select a window of pixel with size mxn with the pixel intensity values ranging from 0 to 255.*
(*ii*) *Split the window into individual cells.*
(*iii*) *For every pixel in a given cell, the pixel is compared with its eight neighbors in a clockwise circular direction.*
(*iv*) *If the pixel value at the center is greater than its neighbor pixel value, then the value is set to zero; otherwise, it will be set to one. An eight-digit binary number will be generated from each window.*
(*v*) *Histogram with dimensions of mxn over the cell is computed, repeated for every combination, and normalized the resultant histogram.*
(*vi*) *Concatenate all the normalized histograms can generate a feature vector.*

(9) $$\mathrm{LBP}=\sum_{i=0}^{p-1}s(ni_{\;}-G_{c})\;2^{i}$$

where s(x) = $\{\begin{array}{l} 1,\;if\;x>0\\ 0,\;otherwise \end{array}$

### 3.4. Deep Network as Features Descriptor

The convolutional neural network (CNN) [21] is the best among the categorized neural networks. CNNs hide layers and perform well in image processing for object detection and recognition tasks. Classifying objects is based on the features provided from the preceded formal layers in the network. Usually, the convolution, pooling, fully connected, and output layers can form a traditional convolutional neural network. A convolution layer is a combination of learning filters whose size is smaller than the input size, and these kernel filters are enforced on input feature maps to obtain the feature map outputs. A pooling layer is also known as the down sampling layer, which will decrease the dimension of features and is categorized into two types of pooling: max pooling and mean pooling. Max pooling reduces the reduction of the actual image to 25 percent by retaining the maximum output area. In contrast, mean pooling helps maintain the average of the output area. A fully connected layer has all the nodes connected from the previous layer. This layer is utilized to converge all the features extracted by the previous layer. The fully connected layer output will provide high purity and integrity features, suitable for the classifier to justify the objects from an image at the output layer. A Softmax regression classifier will be used in most of the convolutional neural networks.

In our proposed system, our CNN architecture has three convolutional layers and two fully connected layers. The first convolutional layer has eight filters of size 3 × 3 and a stride size of 1. It is followed by the batch normalization layer, the activation layer with ReLU (Rectified Linear Unit), and the max-pooling layer with a size of 2 × 2 and a stride of 1. The second convolutional layer has sixteen filters of size 3 × 3 with a stride size of 1, followed by batch normalization, ReLU activation layers, and the max-pooling layer. The third convolutional layer has thirty-two filters of size 3 × 3, followed by batch normalization, and the ReLU activation layer. Then, the first fully connected layer has an output size of 1000 neurons, whereas the second fully connected layer has an output size of 7 neurons. The SVM employs the outputs of the second fully connected layer for classification.

CNN alone can automatically extract features from the images through convolution and pooling layers. Extracting features can reduce unnecessary data from the training database, which builds a speed and better learning model with less machine efforts. In addition to that, we introduced two unsupervised techniques for feature extraction such as Gabor filter and local binary patterns (LBP), which can help in reducing the CNN’s workload in the extraction of the face image characteristics by keeping the internal changes within the class to a minimum value. Due to their tolerance to illumination changes and simplicity in computation, LBP is one of the best texture descriptors to handle the illumination challenges. Gabor has remarkable visual properties such as orientation selectivity and spatial frequency selectivity, which help extract features from the face aligned at different angles. SVM has many advantages in solving pattern recognition, classification and regression problems. Thus, adding the fusion of hand-crafted feature descriptors Gabor and LBP features to the convolutional neural network to yield more robust deep features and replacing the softmax classifier with SVM which helps with more efficient classification.

### 3.5. Support Vector Machine (SVM) Classifier

SVM [18] is considered an important supervised learning method used for classification and regression purposes to increase prediction accuracy and eliminate data overfitting. These support vector machines use the structural risk minimization (S.R.M.) principal formulations. Using this technique, the superiors are projected to the empirical risk minimization (ERM) principle used by a convolutional neural network. S.R.M. minimizes the expected risk upper bound and ERM reduces the training data error rate. This algorithm can compute the optimal hyperplane to separate the classes for a given labeled training data. Every point in the feature’s space that belongs to each class is grouped and separated from the other class by a marginal space.

For the binary classification cases, the training vectors *v_i_* ∈ R^n^, *i* = 1,…*k* and the respective set of ‘*k*’ labels *y_i_* ∈{1, −1} then optimization will be carried out by

(10) $$min_{w,b,\xi \;}(\frac{1}{2}w^{T}w+C\sum_{i=1}^{k}\xi_{i})$$

(11) $$y_{i}\left(w^{T}\Phi \left(v_{i}\right)+b\right)\geq 1-\xi_{i}.\xi_{i}\geq 0,i=1,\ldots .k$$

where $\xi_{i}$ is the error in misclassification for the *i*th vector in training, *w* is the hyperplane normal vector. These vectors are mapped into high dimensional space with function Φ and kernel function *K* ($v_{i},v_{j}$) = $\Phi {\left(v_{i}\right)}^{T}\Phi \left(v_{i}\right)$.

The computation of the support vector machine output can be carried out by

(12) $$\left[\frac{1}{N}\sum_{i=1}^{n}\max\left(0,1-y_{i}\left(w^{T}X_{i}-b\right)\right)\right]+\lambda {\left|\left|w\right|\right|}^{2}$$

As the number of classes in the proposed problem is greater than two, every two classes are trained against each other using the one-vs-one SVM training technique with hinge binary loss function. A convolutional neural network has been constructed for the emotion detection model with fifteen layers in total. The network has three convolutional layers, each with batch normalization and rectified linear activation function (ReLU) layer to prevent negative features. This layer performs element-wise operations and contains a rectified feature map output. Batch normalization [56] is added to the convolutional layers to eliminate the internal covariance transfer phenomenon to obtain better results. The batch normalization layer’s main function is to round up the input data to a mean of zero with a variance of one to improve network convergence and training time. The deep network output is used as input for the support vector machine classifier to predict the driver’s emotions at the classification stage.

## 4. Experimental Results

**Figure 3:**
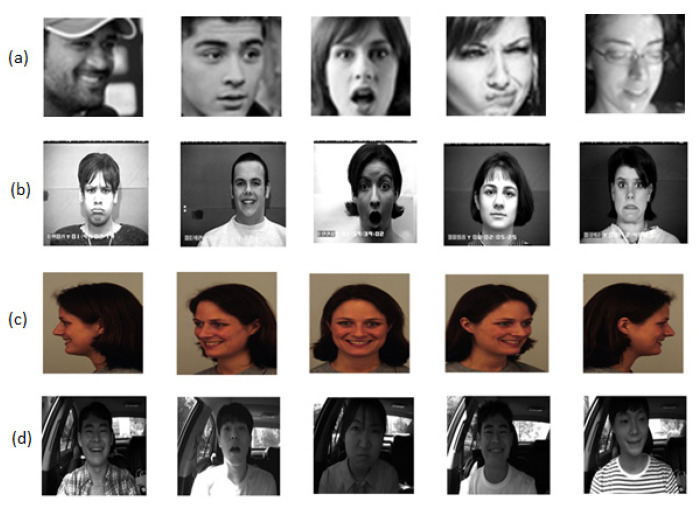
(**a**) FER 2013 Dataset. (**b**) CK+ Dataset. (**c**) KDEF Dataset. (**d**) KMU-FED Dataset.

We evaluate the performance of our method on four well-known publicly available benchmark datasets such as FER 2013 (face expression recognition 2013) [48], CK+ (extended Cohn Kanade) [49], frequently used for facial emotion recognition and KDEF (Karolinska directed emotional face) [50] for direction emotional recognition. Since we are developing a model for driver emotion detection, KMU-FED (Keimyung University Facial Expression of Drivers) dataset [51] is chosen, which has captured images of the driver’s facial emotions with different emotions in a driving scenario in real-time. Figure 3 demonstrates the samples of different expressions from the dataset used. The details of the experiments and results will be expressed in the following sections.

### 4.1. Implementation Details

In our experiment at the pre-processing stage, a Gaussian filter with a standard deviation of 0.5 and a window size of 3 × 3 is used for smoothening. The median filter of a window size of 3 × 3 is used for noise removal. Histogram equalization and a wiener filter with a 3 × 3 window size is utilized for image enhancement and adaptive noise removal. For detection of facial features, different cascade classification models are trained with image sizes (12 18), (15 18), (15 25) for detecting the facial features such as eyes, nose, and mouth, with a scale factor of 1.1 and a detection threshold value of 4. We have selected the wavelength of 4 and 90° orientation for the Gabor filter to extract features manually along with LBP. These extracted features are fused and fed into CNN for more robust features. The dataset was split into an 80:20% ratio for training and testing in all experiments. The parameter settings for training CNN in our proposed approach on all the four databases are shown in Table 1. We have chosen a stochastic gradient descent optimizer with momentum having learning rate of 0.001 with cross entropy as loss function, ReLU as the activation function and trained to a maximum number of 100 epochs. Later for classification, we selected one vs. one type SVM with a linear kernel with hinge loss function and SGD solver as shown in Table 2. All the experiments to develop our hybrid model for driver emotion detection have been conducted using MATLAB in a system environment that includes a ninth Generation Intel Quad-Core Processor i5-9300H with 12 G.B. of RAM and NVIDIA GeForce GTX 1650 GPU running Microsoft Windows 10 operating system.

### 4.2. Dataset description

#### 4.2.1. FER 2013 Dataset

It is a facial expression recognition 2013 [48] dataset that consists of 35,887 images having a resolution of 48 × 48 pixels. Using Google, the images in the dataset are requested and collected according to key emotions. The images are captured in a wild environment, including different poses, blurring, and occlusions, making recognition more challenging. Different facial poses, illuminations variations, and occlusions such as sunglasses and hair are involved in this dataset images. Samples of the FER 2013 dataset are shown in Figure 3a.

#### 4.2.2. CK+ Dataset

This is popularly called the Extended Cohn-Kanade dataset [49], which is expanded from the Cohn-Kanade dataset, published in 2010. It consists of 123 subfolders and 593 image sequences with different facial expressions. It is one of the widely used benchmark datasets in facial expression recognition. One hundred and twenty-three participants with an age range from 18 to 50 are involved, of which 81% are Euro-American, 13% African American, and the remainder are 6%. The dataset images are with a resolution of 640 × 480 and 640 × 490 pixels contain a facial shift from a neutral expression to the targeted peak expression—image samples of CK+ dataset are s shown in Figure 3b.

#### 4.2.3. KDEF Dataset

This is called as Karolinska Directed Emotional Face (KDEF) [50] dataset and contains 4900 images of human emotional facial expressions captured from 35 male and 35 female individuals at five different angles: A half left rotated, full left rotated, half right rotated, full right rotated, and frontal view images. These images in the dataset are with a resolution of 562 × 762 pixels. For medical and psychological research purposes, this dataset was developed earlier. However, most emotion recognition works use this dataset for experimental research—image samples of the KDEF dataset are shown in Figure 3c.

#### 4.2.4. KMU-FED Dataset

We have opted for the Keimyung University Facial Expression of Drivers (KMU-FED) dataset [51] to evaluate our proposed hybrid model for driver emotion detection. This dataset contains the images of drivers having various facial expressions while driving in real-time. Near-infrared (NIR) camera installed on either dashboard or steering wheel is used to capture the driver’s emotions. There are 55 image sequences generated from 12 subjects involved with illumination conditions such as left light, front light, backlight, and right light, with partial occlusions of sunglasses and hair. The images in this dataset have a resolution of 1600 × 1200 pixels—image samples of the KMU-FED dataset are shown in Figure 3d.

### 4.3. Performance Evaluation

#### 4.3.1. Experiments on FER 2013 Dataset

To verify the effectiveness of the proposed hybrid model for driver emotion detection, we have compared our model performance with recent state-of-the-art FER methods including CNN-MNF [37], CCNN-BOVW-SVM [36], KCNN-SVM [39], VCNN [30], EXNET [31], Deep-Emotion [32] and IRCNN [42]. Table 3 reports our experimental results and shows the comparison with these methods. In Table 3, some of the works have used the deep neural network approach alone and the others have used combination of traditional machine learning with deep neural network as hybrid approach. In hybrid approaches such as CNN-MNF [37], CCNN-BOVW-SVM [36], KCNN-SVM [39], and IRCNN [42], they have used multiple convolutional neural network architectures for feature extraction and fused these features for SVM classification. In the deep neural network approaches such as VCNN [30], EXNET [31], and Deep-Emotion [32], they have merged different network architectures which involve either attention mechanism on facial-region-containing features or used parallel feature extraction. For both the cases, our method significantly outperforms all others with an achieved accuracy of 84.4% vs. the previous best of 80.3% with the hybrid approach of integrated clustered samples with CNN and SVM [39], which surpass it by 4.1%.

#### 4.3.2. Experiments on CK+ Dataset

We have compared our proposed hybrid model with recent state-of-the-art FER methods, including Inception-Resnet and LSTM [35], DCMA-CNN [27], WRF [22], LMRF [24], VGG11+SVM [40], DNN+RELM [43], LBP+ORB+SVM [25], and MDNETWORK [33] on the CK+ dataset. These works adopted machine learning algorithms and deep neural networks in combined manner or individually used. Among them, WRF [22], LMRF [24], and LBP+ORB+SVM [25] have created their FER models using machine-learning-based feature extraction methods and classification mechanisms. In the remaining works, Inception-Resnet and LSTM [35], DNN+RELM [43], VGG11+SVM [40] have used both deep neural networks and classifiers along with handcrafted features extracted by machine learning. DCMA-CNN [27] and MDNETWORK [33] were implemented using multi branch convolutional neural networks. Our proposed hybrid approach outperforms the state-of-the-art models with an achieved accuracy of 95.1% listed in Table 4, except for MDNETWORK [33] which is 1.1% greater than our accuracy.

#### 4.3.3. Experiments on KDEF Dataset

Our proposed hybrid approach is compared with recent state-of-the-art FER methods, including MULTICNN [28], HDNN [38], RTCNN [29], ALEXNET+LDA [41], MSLBP+SVM [23], DL-FER [34], and RBFNN [26], which used either machine learning methods alone without using CNN or a combined model of CNN and SVM methods on the KDEF dataset. Table 5 reports our experimental results and shows the comparison with these methods. In Table 5, MSLBP+SVM [23], RBFNN [26] used machine learning methods for feature extraction and classification, whereas DL-FER [34], MULTICNN [28] and RTCNN [29] used deep neural networks alone. ALEXNET+LDA [41] and HDNN [38] used the framework of combining deep neural network for feature extraction and machine learning techniques for classification. As shown in Table 3, it can be seen that our proposed hybrid approach achieved the best performance with an accuracy of 98.5%, among the methods compared. The obtained accuracy surpasses the recent machine-learning-based work, i.e., RBFNN [26] by 9.7% and recent deep-neural-network-based work, i.e., DL-FER [34], by 1.9%.

#### 4.3.4. Experiments on KMU-FED Dataset

Our proposed hybrid approach is compared with recent state-of-the-art methods including WRF [22], FTDRF [24], d-RFs [24], SqueezeNet [24], MobileNetV3 [24], LMRF [24], CCNN [44], and VGG16 [45], which used the KMU-FED dataset. These works utilized deep-neural-network-based approaches as well as various types of dense random-forest (DRF)-based approaches. Table 6 reports our experimental results and shows the comparison with these methods. Deep network approaches such as CCNN [44], SqueezeNet [24], MobileNetV3 [24], VGG16 [45] have used pre-trained network architectures for feature extraction and classification of driver expressions from the input images. Dense random-forest-based approaches such as WRF [22], FTDRF [24], d-RFs [24], and LMRF [24] have used a smaller number of decision trees compared with actual random forests, which are completely machine-learning-based algorithms. As shown in Table 6, it can be seen that our proposed hybrid model approach achieves the best performance among the methods compared with an accuracy of 98.6%, which surpasses the recent machine-learning-based work, i.e., LMRF [24] by 3.5%, and also outperforms the recent deep-neural-network-based work, i.e., VGG16 [45] by 4.4%.

### 4.4. Emotion Recognition Results

**Figure 4:**
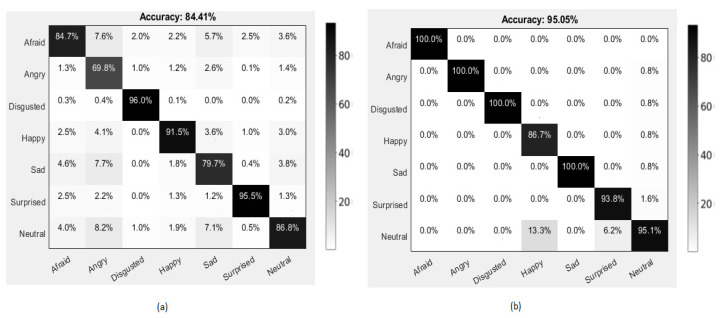

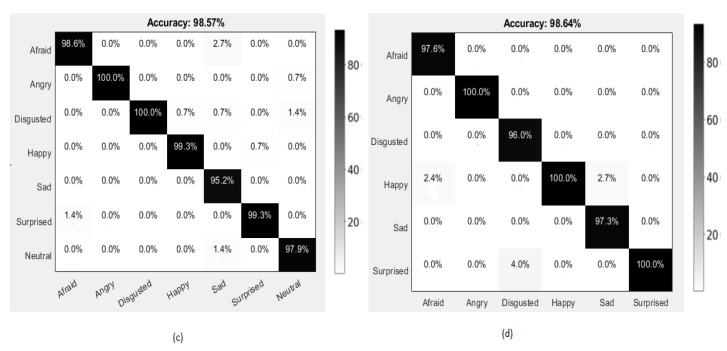
Confusion matrices of the proposed method with accuracy (%) using (**a**) F.E.R. 2013 dataset, (**b**) CK+ dataset, (**c**) KDEF dataset, and (**d**) KMU-FED dataset.

For the comparative analysis, we have shown the confusion matrices of our proposed approach on FER2013, CK+, KDEF, and KMU-FED datasets in Figure 4. Figure 4a represents the emotion recognition accuracies on the FER2013 dataset, which has different occlusions such as hair, sunglasses, and low-intensity illumination conditions in which surprise and disgusted expressions are predicted with more than 95% accuracy. Neutral and afraid expressions are predicted with ≥85% accuracy. In contrast, angry expressions are predicted with low accuracy. Figure 4b shows that emotion recognition accuracies on CK+ dataset in which afraid, angry, disgusted and sad expressions are predicted with zero error. Surprised and neutral expressions are predicted with ≥90%, with the least prediction accuracy on happy expression. Figure 4c represents emotion recognition accuracies of the KDEF dataset in which facial images with expressions are captured in five different rotation angles. It is quietly challenging to recognize the expression from them. Angry and disgusted expressions are predicted with zero error rate; happy and surprised expressions are predicted with ≥99% accuracy. Sad, afraid, and neutral expressions are predicted with ≥95% accuracy from the KDEF dataset. Figure 4d represents the KMU-FED dataset in which the driver’s facial image expressions are captured in the real-time driving environment under different illumination changes with hair and sunglasses occlusions. From this dataset, angry, happy, and surprised expressions are predicted with zero error rate, and the remaining expressions are predicted with ≥96% accuracy. From all the above confusion matrices, we can observe that angry expression is predicted in most of the datasets involved, which is crucial to monitor while driving. Fearful expressions are predicted with ≥97% accuracy in most of the datasets. The surprise expression is predicted with ≥93% in all of the datasets involved with different environments.

### 4.5. Limitations

New developments have been made in the field of deep learning such as Vision Transformer ViT [57], which uses a self-attention mechanism to capture global contextual information. This technique has some limitations and disadvantages, especially in real-time applications, such as driver’s emotion detection. As Transformers are mainly designed for Natural Language Processing applications, data patch orders are not considered during training or testing. For computer vision tasks, the data patch’s order is very important to reserve the semantic meaning of the objects [57]. To solve this problem in Transformers, an extra encoder must be added to reserve data order and sequence, which rapidly increases computational complexity for both training and testing. Moreover, the attention mechanism used in the Transformers can only perform feature aggregation and an extra layer of feedforward network needs to be added to calculate required transformations. All these extra layers are considered to add more time and computational complexity to Transformer models.

On the other hand, convolutional-based models can carry out both aggregation and transformation in the same layer, which can be considered more computationally efficient for real-time applications [58]. For these reasons, new techniques are still under investigation to find an optimized Transformer architecture suitable for real-time vision applications. These new techniques are trying to overcome these problems, but they are facing other challenges such as difficult in training [59], accuracy loss due to binarization [60], slow convergence, and poor performance, while tracking small objects due to the exponential increase in computational complexity of the self-attention operator.

For these reasons, the CNN model with traditional techniques of computer vision and machine learning is adopted in this research to solve this important drive safety application. Although the proposed approach achieved its primary objectives in detecting drivers’ emotions from facial images, it has some limitations that need to be addressed in the future. One of these limitations is detecting high-intensity levels in the driver’s emotions that occur and change vividly while driving on mountain cliffs, deep valleys, and ridges. Another limitation of the proposed technique is driving with masks, as all images used in the testing process are for persons without masks, and there is no sufficient number of face images with masks for facial expression applications. All these limitations and challenges will be included as part of the extended work of this research.

## 5. Conclusions

Current facial expression recognition research has mostly focused on creating a model by involving a convolutional neural network for feature extraction and support vector machine for classification purposes. Despite that, some models are using dedicated machine learning methods for feature extraction and classification. However, they are still limited, along with the existing challenges such as occlusions, illumination, and pose variation. These challenges have been addressed using the proposed technique. As stated, the proposed technique has presented a new hybrid methodology in which a convolutional neural network is used with a support vector machine as a classifier for improving prediction accuracy. Unlike previous techniques, the paper has employed dedicated computer vision feature extraction methods such as local binary patterns and Gabor filters that are fused to obtain new features. The new extracted features are fed to the convolutional neural network for more robust features, and further classification is carried out using a support vector machine. The presented technique has achieved an accuracy of 84.41%, 95.05%, 98.57%, and 98.64% on FER2013, CK+, KDEF, and KMU-FED datasets, respectively. For future work, enhanced architecture deep networks can be used with fused classifiers and combined with decision-making techniques to enhance the classification capability in prediction, which will help in vision-based surveillance applications.

## Figures and Tables

**Table 1:** Parameter settings used to train our CNN model on all four databases.

Databases	Parameters	Settings
	Image Size	256 × 256
	Optimizer	Stochastic Gradient Descent (S.G.D.)
CK+,	Loss Function	Cross Entropy
FER 2013,	Activation Function	ReLU
KDEF,	Batch Size	128
KMUFED	Learning rate	0.001
	Epochs	100
	Momentum	0.9
	Validation Frequency	30

**Table 2:** Parameter Settings used to train our SVM model on all four databases.

Databases	Parameters	Settings
	Objective Function	Hinge loss
CK+,	Solver	SGD
FER 2013,	Kernel	Linear
KDEF, KMUFED	Type	One-vs-one

**Table 3:** Comparison of proposed approaches with the state-of-the-art methods on FER 2013 Database.

Comparison Methods	Accuracy (%)
CNN-MNF [37]	70.3
CNN-BOVW-SVM [36]	75.4
KCNN-SVM [39]	80.3
VCNN [30]	65.7
EXNET [31]	73.5
Deep-Emotion [32]	70.0
IRCNN-SVM [42]	68.1
GLFCNN+SVM (Our Proposed Approach)	84.4

**Table 4:** Comparison of proposed approaches with the state-of-the-art methods on CK+ Database.

Comparison Methods	Accuracy (%)
Inception-Resnet and LSTM [35]	93.2
DCMA-CNN [27]	93.4
WRF [22]	92.6
LMRF [24]	93.4
VGG11+SVM [40]	92.2
DNN+RELM [43]	86.5
LBP+ORB+SVM [25]	93.2
MDNETWORK [33]	96.2
GLFCNN+SVM (Our Proposed Approach)	95.1

**Table 5:** Comparison of proposed approaches with the state-of-the-art methods on KDEF Database.

Comparison Methods	Accuracy (%)
MULTICNN [28]	89.5
HDNN [38]	96.2
RTCNN [29]	88.1
ALEXNET+LDA [41]	88.6
MSLBP+SVM [23]	89.0
DL-FER [34]	96.6
RBFNN [26]	88.8
GLFCNN+SVM (Our Proposed Approach)	98.5

**Table 6:** Comparison of proposed approaches with the state-of-the-art methods on KMU FED Database.

Comparison Methods	Accuracy (%)
WRF [22]	94.0
FTDRF [24]	93.6
d-RFs [24]	91.2
SqueezeNet [24]	89.7
MobileNetV3 [24]	94.9
LMRF [24]	95.1
CCNN [44]	97.3
VGG16 [45]	94.2
GLFCNN+SVM (Our Proposed Approach)	98.6

## Data Availability

Not Applicable.

## Abbreviations

The following abbreviations are used in this manuscript:

AAA	American Automobile Association
ADAS	Advanced Driver Assistance Systems
CNN	Convolutional Neural Network
DL	Deep Learning
E.C.G	Electrocardiogram
E.D.A	Electrical Dermal Activity
E.E.G	Electroencephalogram
FER	Face Expression Recognition
LBP	Linear Binary Pattern
ML	Machine Learning
P.P.G	Photoplethysmography
ReLU	Rectified Linear Unit
ROI	Region of Interest
S.G.D	Stochastic Gradient Descent
SVM	Support Vector Machine
